# The Impact of BOLD‐Induced Linewidth Modulation on Functional ^1^H MRS Analysis

**DOI:** 10.1002/nbm.70392

**Published:** 2026-09-20

**Authors:** Martin Wilson, Simon M. Finney, William T. Clarke

**Affiliations:** ^1^ Centre for Human Brain Health and School of Psychology University of Birmingham Birmingham UK; ^2^ Oxford Centre for Integrative Neuroimaging (OxCIN), FMRIB, Nuffield Department of Clinical Neurosciences University of Oxford Oxford UK

## Abstract

Functional MRS can measure the neurometabolic response to neuronal activation, therapeutic interventions and changes in physiology. Substantial technical challenges currently present a barrier to reproducible findings and broader adoption by the neuroscientific community. One such challenge is the conflation between genuine metabolic changes and bias caused by subtle spectral lineshape changes associated with the BOLD response. Previous studies have demonstrated an approximately 1% bias for glutamate estimates at 7 T based on experimentally acquired data and a single conventional fitting algorithm. In this study, we use synthetic MRS data to estimate the bias for two conventional fitting methods (LCModel and ABfit‐reg) at 3 and 7 T and evaluate the efficacy of dynamic lineshape adjustment, during preprocessing and fitting analysis stages, to reduce bias. Using the same dataset, we also explore the potential bias in 2D fitting approaches, comparing several fitting models implemented in FSL‐MRS. The two conventional fitting methods, without explicit linewidth correction, had similar levels of bias (~1% for glutamate)—in good agreement with previous experimental studies at 7 T. Lineshape changes from the BOLD response cause similar bias in conventional and 2D fitting packages for 3 and 7 T data, resulting in an overestimation of metabolic changes associated with neuronal activation. This bias may be significantly reduced (< 0.2%) by incorporating a BOLD linewidth matching step for conventional analysis or by direct modelling for 2D analysis. We therefore recommend explicit BOLD lineshape correction or modelling for future task‐based fMRS studies at 3 T and above.

## Graphical abstract

Functional MRS studies face a technical challenge: BOLD‐related lineshape changes can be mistaken for genuine metabolic changes, causing ~1% glutamate estimation bias at 3 and 7 T across conventional (LCModel, ABfit‐reg) and 2D fitting methods. Using synthetic data, this study shows that explicit BOLD linewidth correction—via dynamic lineshape adjustment or direct modelling—reduces this bias to under 0.2%. We recommend such correction for all future task‐based fMRS studies.

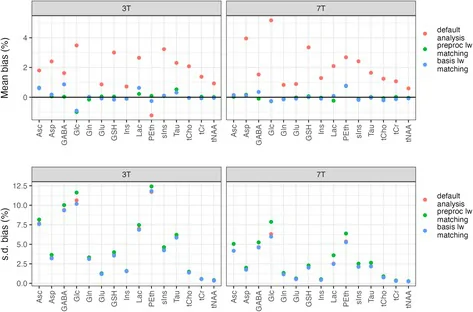

## 1 Introduction

Functional magnetic resonance spectroscopy (fMRS) is a noninvasive contrast that measures dynamic changes in metabolite signal associated with neuronal activation [1, 2]. Early studies demonstrated an increase in visual cortex lactate in response to a prolonged period (~10 min) of visual stimulation [3], and these findings have been replicated by various research groups across multiple acquisition platforms and analysis methodologies [4, 5, 6, 7, 8, 9, 10, 11]. More recently, methodological advances have established associated changes in glutamate, glucose and aspartate [4, 6, 9, 10], providing novel information on the roles of the aerobic glycolytic pathway, malate–aspartate shuttle and tricarboxylic acid cycle in neural activity. Alternative paradigms, including pain [12, 13, 14], motor tasks [15, 16, 17] and event‐related designs [18, 19], have also demonstrated various changes in metabolite levels in response to brain activation.

Whilst fMRS provides novel molecular insight into localised brain activation [20], the technique is technically challenging to perform. This is primarily due to the relatively low signal strength of the metabolites compared with spectral confounds, such as thermal noise, and the insufficient suppression of water and lipid signals. The positive BOLD effect presents a further counterintuitive confound by inducing a small reduction in spectral linewidth during periods of increased brain activation [21, 22]. In principle, established spectral fitting methods [23] model lineshape changes, implying they should not influence metabolite levels. However, comparisons of fitting models have found biased estimations of lineshape in some packages [24] and fMRS studies at 7 T have demonstrated how minor spectral narrowing biases dynamic metabolite estimates [4, 25]. More broadly, the impact of imperfect lineshape estimation on metabolite estimates is well established for conventional [26, 27, 28, 29, 30] and edited MRS [31]. This bias presents a significant concern for fMRS interpretation, as genuine task–induced changes in metabolite levels are generally concurrent with the BOLD effect, and therefore, BOLD‐induced fitting bias may be erroneously attributed to genuine changes in metabolite levels.

At the time of writing, the fMRS community is yet to reach consensus on precisely which experimental conditions warrant explicit correction of the BOLD‐induced bias, with prevailing opinion suggesting these effects only need to be ameliorated at field strengths of 7 T and above. With this opinion presumably being motivated by the BOLD effect's linear dependence on field strength [32]. The most widely used correction involves applying Lorenzian linebroadening to the spectra acquired during the task‐active portion of the experiment to match those acquired in the rest or baseline phase [4, 10, 17, 33, 34]. We will refer to this approach as ‘Lorentzian lineshape matching’ in this paper. Additionally, white noise may be added to the artificially broadened spectra to eliminate any potential SNR bias between the rest and task phases [30]. Both these steps are performed during the preprocessing phase of analysis before spectral fitting [35].

In this study, we quantify the BOLD‐induced bias on metabolite estimates and assess several strategies to mitigate the effect. In contrast to previous studies, based on in vivo data [4, 25], we use synthetic MRS to precisely isolate the BOLD‐induced bias from other potential confounds—such as participant movement or scanner drift [36]. Furthermore, our analysis goes beyond conventional 1D fitting at 7 T to also include 3 T and 2D dynamic fitting [37, 38, 39], covering a wider range of contemporary fMRS methodologies. We also address the effect of noise colouring introducing bias during fitting when the Lorentzian lineshape matching approach, also known as apodisation, is used.

## 2 Methods

The generation and analysis of synthetic MRS are currently the only approach to offer ‘ground‐truth’ knowledge of the dynamic spectral changes associated with fMRS. Therefore, our methodology uses simulation of the expected dynamic lineshape change associated with the BOLD response to a prolonged task—currently the most common fMRS experimental design. Simulation parameters, such as SNR, linewidth and metabolite levels, are designed to match those from previously published studies acquired at field strengths of 3 and 7 T. In contrast to experimentally acquired MRS, simulated metabolite levels will not change dynamically; therefore, any task‐associated change in metabolite levels may be directly interpreted as BOLD‐induced bias; that is, the expected ground truth result is for no metabolite concentration change.

### 2.1 Synthetic Data

The list of metabolite, macromolecule and lipid signals and concentrations used to generate the synthetic MRS data is given in Table S1. Metabolite chemical shift and J‐coupling values were taken from Govindaraju et al. [40, 41]; broad lipid and macromolecule signal parameters were taken from Table 1 in Wilson et al. [42]. Metabolite concentration values, emulating visual cortex, were taken from Table 1 in Bednarik et al. [4]. Alanine and Glycine were not listed in this table and therefore did not contribute to the synthetic MRS data; however, both metabolites were included in the subsequent fitting basis set.

**TABLE 1:** Simulation parameters for 3 and 7 T synthetic datasets.

	3 T	7 T
Single shot SNR	45	80
TR (seconds)	2.5	5
Number of excitations	640	320
Applied Gaussian broadening (Hz)	6	11
BOLD Lorentzian linewidth change (Hz)	0.2	0.46

Metabolite signals were simulated for the semi‐LASER sequence [43] with an echo time of 28 ms, acquired over 2048 complex points sampled at a frequency of 6000 Hz. The total scan time was 26 min and 40 s. Table 1 lists simulation parameters that differ between 3 and 7 T based on typical values for visual cortex acquisitions reported in the literature [4]. Example spectra are plotted in Figure S1. No additional signals (such as residual water or lipids) were included to model baseline deviations.

**FIGURE 1:**
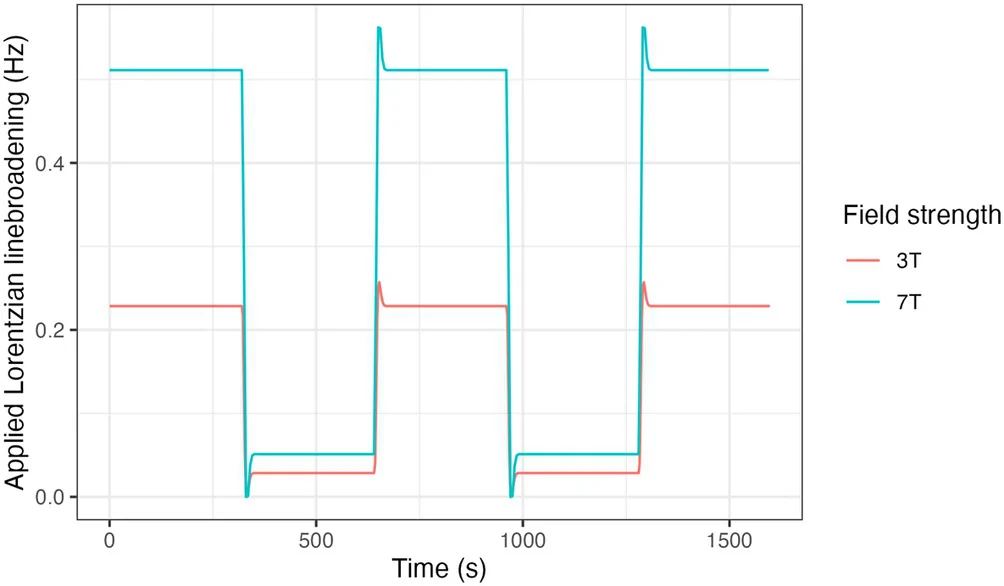
Applied Lorentzian linebroadening to simulate the spectral BOLD effect at 3 T and 7 T.

A simple prolonged block design was assumed to generate the BOLD linewidth changes. Five blocks of equal duration (320 s) were ordered as REST–TASK–REST–TAST–REST, with the TASK blocks corresponding to a reduction in Lorentzian linewidth of 0.2 and 0.46 Hz at 3 and 7 T respectively, based on previous studies of fMRS visual stimulation [4, 22]. In practice, Lorentzian linebroadening was applied to the REST blocks and its contribution reduced in the TASK regions assuming a BOLD response derived from the widely used double gamma model [44]. Figure 1 shows the applied Lorentzian linebroadening for both field strengths, with the notable feature that a constant value was added to both responses to ensure all values were nonnegative to avoid numerical instability. More rapid changes in the simulated BOLD response are observed at the beginning and end of the task (known as the ‘initial overshoot’ and ‘post‐stimulus undershoot’ respectively) consistent with standard modelling assumptions widely used for fMRI analysis. These features may appear unusually pronounced due to the relatively prolonged stimulation period compared with typical fMRI paradigms. The BOLD linewidth changes (including the initial overshoot and post‐stimulus undershoot) shown in Figure 1 were used for both simulation and analysis.

The full dataset consisted of 256 synthetic fMRS scans (128 at 3 T and 128 at 7 T) with additive Gaussian distributed complex noise to match the single‐shot SNR values given in Table 1. This number of scans was chosen to be large enough to accurately estimate relevant bias from typical fMRS experiments, which generally consist of less than 20 participants. Each fMRS scan was designated a subject ID (e.g., sub‐001, sub‐002 …) stored in NIfTI‐MRS format [45] and organised according to BIDS specification [46].

### 2.2 Lorentzian Lineshape Matching

The most common method to reduce BOLD line‐narrowing–induced fitting bias implements a data preprocessing step where Lorentzian linebroadening is applied to data acquired during the TASK phase of the experiment to match the REST phase. In this study, we have also applied the Lorentzian lineshape matching preprocessing step to the synthetic data to evaluate its efficacy in reducing fitting bias. In addition to applying Lorentzian linebroadening to maintain linewidth consistency across the dynamic scans (based on the known curves shown in Figure 1), we also add additional complex Gaussian noise to maintain SNR consistency—as performed by Bednarik et al. [4]. Figure S2 illustrates this procedure, with comparison between parts B and C showing the lineshape matching step and C and D showing the noise matching step. Analysis incorporating this approach will be denoted ‘preproc lw matching’ in subsequent plots.

Before fitting, Lorentzian lineshape matching (as described above) was applied to a copy of the data for the ‘preproc lw matching’ analysis, whilst the original un‐matched was used for all other analyses (‘basis lw matching’ and ‘default analysis’).

### 2.3 Conventional Fitting and Preprocessing

Dynamic frequency and phase alignment is a standard preprocessing step for fMRS; therefore, the RATS method [47] was applied to each dataset. Averaging of temporal dynamics was also applied to reduce the original, unaveraged data down to five blocks (REST, TASK, REST, TASK, REST). Two alternative averaging schemes, two blocks (REST, TASK) and 10 blocks (REST, REST, TASK, TASK, REST, REST, TASK, TASK, REST, REST) were also assessed.

Two fitting packages were used:

1. LCModel [23], as this package has been reported to exhibit BOLD‐induced bias [25] and is widely used for fMRS analysis,
2. ABfit‐reg [48], to explore if bias differs significantly between conventional fitting algorithms.

Default fitting options were used for both fitting packages in all analyses, and the same basis set used for synthetic MRS generation was used for spectral fitting (Table S1).

As an alternative to the conventional preprocessing lineshape matching step, we performed an additional analysis where the linewidth of the fitting basis set was adjusted (prior to subsequent adjustment within the fitting method) to compensate for the change arising from the predicted BOLD response. For example, in the 3 T data analysis, 0.2 Hz Lorentzian linebroadening was applied to the basis set for the REST blocks, but not the TASK blocks. This was performed as an attempt to equalise the magnitude of any initial fitting discrepancy (and therefore potential bias) between the REST and TASK states. Analysis incorporating this approach is denoted ‘basis lw matching’ in subsequent results. The direct adjustment of basis‐set linewidth prior to conventional fitting as a means of improving accuracy has been reported previously [28, 49, 50]; however, to the best of the authors' knowledge, the present study is the first to apply this technique specifically to reduce BOLD bias in fMRS.

Synthetic MRS generation, preprocessing and conventional fitting were performed using version 3.9.0 of the spant MRS analysis package [51].

### 2.4 Dynamic Fitting

Dynamic fitting, the simultaneous modelling of the spectral and (functional) temporal domains, was also assessed as a method to minimise line‐narrowing bias. This assessment was carried out using FSL‐MRS [37, 52] version 2.4.10, on the same data (simulated as described above), and using the same fitting basis sets. Four different temporal models were investigated, each modelling the time dependance of metabolite concentrations using a GLM with three regressors: a ‘constant’ term and two rectangular functions aligned with the periods of BOLD‐induced line‐narrowing. The models differed in their treatment of the time dependance of Lorentzian linebroadening term (the FSL‐MRS ‘gamma’ parameter):

1. Fixed Model: The fixed model imposed a constant, but optimisable value of Lorentzian broadening for all timepoints.
2. Variable Model: The variable model estimates an independent Lorentzian broadening for each time point.
3. Exact Model: The exact model imposes the true time‐variable broadening used to simulate the data (Figure 1), but the amplitude of the broadening is estimated directly from the data.
4. Correct Model: The correct model imposes the true time‐variable broadening at a scale set to that used to simulate the data.

For the exact model, the single linewidth scaling parameter was initialised using a Tobit (censored) regression to mitigate the effect of zero‐valued Lorentzian linewidth estimates from single noisy transients. All other model parameters (frequency shift, zero‐order and first‐order phase, Gaussian line broadening) were fitted with a single, optimisable, but time‐invariant value. The FSL‐MRS baseline was disabled to match the simulated data model. The four dynamic models are provided in the linked Github repository https://github.com/wtclarke/fmrs_bold_dynamic_fitting in the dyn_models directory (dyn_models/config_correct_lb.py, dyn_models/config_exact_lb.py, dyn_models/config_fixed_lb.py, dyn_models/config_variable_lb.py).

The FSL tool FLAMEO, which implements multilevel linear modeling for group analysis using Bayesian inference [53], was used through FSL‐MRS's *fmrs_stats* tool to calculate single‐group average statistics for all metabolites. The two blocks of line‐narrowing were combined as an equally weighted first‐level contrast. The group variance was modelled with fixed effects (runmode = fe) as the simulated data include no intersubject variance. The results were calculated as the average estimated change in concentration during the two line‐narrowing periods. The FLAMEO estimated group effect, the standard deviation and Cohen's *d* are reported for major metabolites. Note that all standard deviations (conventional and dynamic) are calculated as the standard deviation of the per‐subject bias; that is, for the dynamic fitting, the reported standard deviation is calculated on the first‐level (per‐subject) bias.

### 2.5 Fitting Approaches

Lineshape and baseline assumptions are known to have a potentially strong influence on analyses, and we therefore outline the principal differences between LCModel, ABfit‐reg and FSL‐MRS below. LCModel and ABfit‐reg both use penalised splines to model the baseline in the frequency domain. Preliminary fits are performed with different levels of baseline flexibility, and the appropriate level is chosen based on the Akaike's information criterion (ABfit‐reg) or confidence region boundaries (LCModel). In this work, for conventional fitting, FSL‐MRS used a first‐order polynomial baseline. For dynamic fitting, FSL‐MRS was used with no baseline model (baseline = ‘off’).

ABfit‐reg allows Lorentzian broadening to be applied to individual metabolite signals (to model T2 variation) and applies a common Gaussian linebroadening to all signals with an additional parameter to model peak asymmetry [54]. LCModel also incorporates individual T2 adjustment but models the common lineshape as a set of convolution coefficients. A smoothness penalty is applied to these coefficients to prevent unrealistic lineshape estimation arising from overfitting. FSL‐MRS implements a voigt lineshape model, that is allowing both Lorentzian and Gaussian broadening. By default, and used in this work, all basis spectra are broadened by the same amount; that is, there is a single Lorentzian and Gaussian parameter. FSL‐MRS does not use any regularisation in its lineshape model.

### 2.6 Bias in Fitting Apodised Data

Lorentzian lineshape matching, the most widely implemented correction in the literature, applies Lorentzian linebroadening—equivalent to an apodisation filter—to the time‐domain FID. The application of this filter affects both the signal and noise in the FID. The noise in the unfiltered FID and the equivalent spectrum (after Fourier transform) is complex, zero‐mean, independent and identically distributed (i.i.d.) Gaussian. In the spectral domain, application of the time domain filter will correlate noise in adjacent spectral bins. For a known filter, the form of this correlation can be derived exactly and is done so for the exponential apodisation (linebroadening) filter in Appendix A.

MRS fitting software typically implements an ordinary least‐squares (OLS) optimisation, which assumes i.i.d. noise. As such, the noise correlation introduced by apodisation could bias fitting results. Optimisation methods such as generalised least squares (GLS) may be used to account for the known noise correlation. To explore mitigation of apodisation‐induced fitting bias, we use an asymptotic framework to predict the leading‐order bias and variance using both OLS and GLS for an idealised fitting problem—fitting a single, Lorentzian (FWHM = 5 Hz), on‐resonance peak. Details of the asymptotic framework are presented in Appendix A. A validation of the asymptotic solutions via comparison to Monte Carlo simulations is presented in Figures S3–S5.

## 3 Results

### 3.1 Conventional Analysis

**FIGURE 2:**
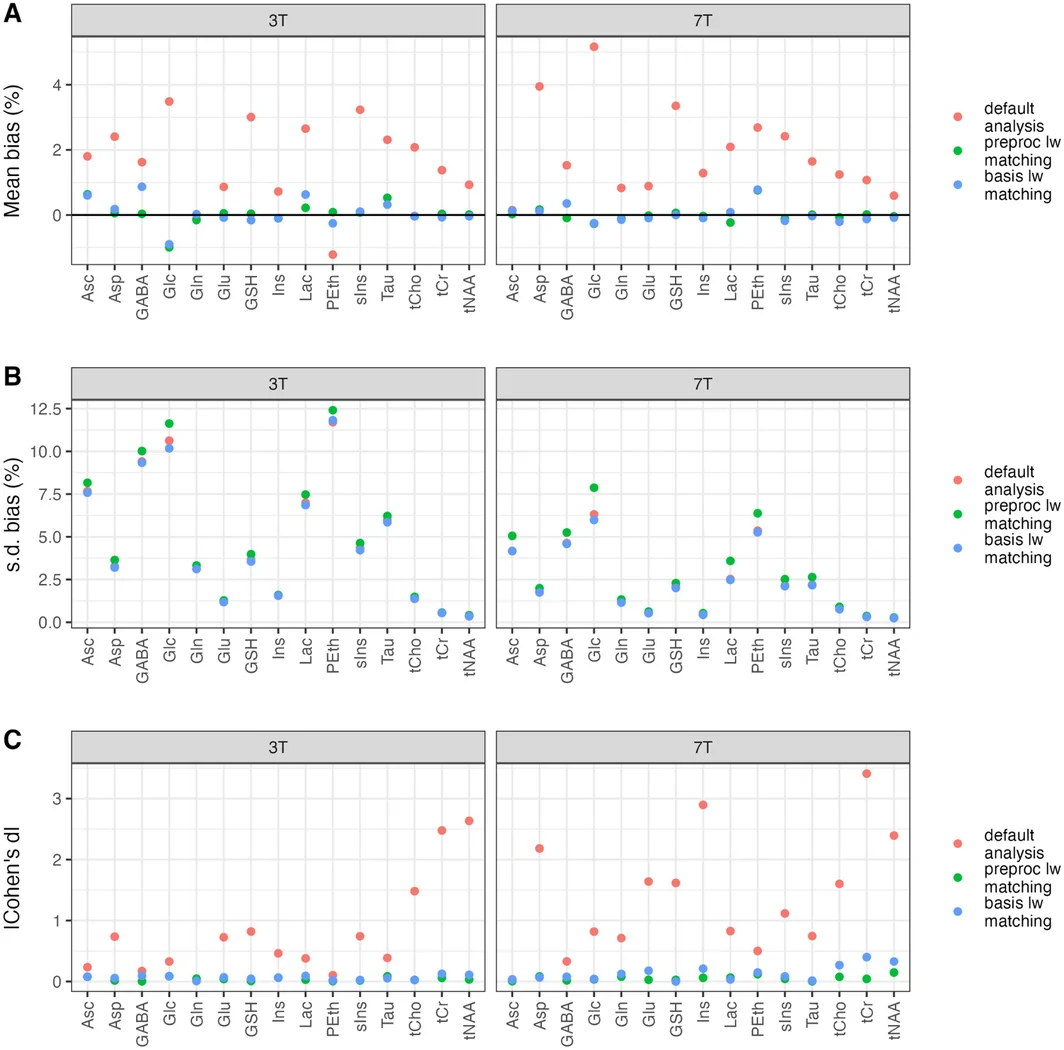
BOLD lineshape–induced bias for commonly measured metabolites at 3 T and 7 T. The ABfit‐reg analysis method was used to analyse five dynamic blocks (REST–TASK–REST–TASK–REST), and three analysis approaches were compared. Bias was calculated by evaluating the mean of the TASK blocks as a percentage change from the mean of the REST blocks on a participant by participant basis.

Figure 2A shows the distribution of bias across a number of commonly measured metabolites with, and without, explicit BOLD lineshape modelling using the ABfit‐reg fitting method. The default analysis bias ranged between approximately −1% to 5%, with glucose being the metabolite most susceptible to bias at 3 and 7 T. Glutamate and lactate are the metabolites most commonly associated with change in fMRS, and these exhibited biases of around 1% and 2% respectively for the default analysis. Preprocessing and basis‐based BOLD linewidth matching approaches both strongly reduced bias for each metabolite by a similar amount. Standard deviations in the bias are shown in Figure 2B, with the preprocessing‐based BOLD linewidth approach showing a slight increase over the basis and default approaches. This increase in variance is likely due to the artificially added noise samples used to match the spectral SNR between the REST and TASK conditions.

An equivalent analysis to Figure 2 for the LCModel and FSL‐MRS fitting methods is shown in Figures S7 and S8, respectively, for comparison with ABfit‐reg. In general, the agreement between the ABfit‐reg and LCModel methods is very high, with the biggest differences in biases being restricted to the more poorly determined metabolites such as phosphoethanolamine. Furthermore, the standard deviation of biases (Figure 2B vs. Figure S7B) shows a very similar pattern across metabolites, with ABfit‐reg showing a small but consistent reduction in standard deviation of around 2.5% for each metabolite. FSL‐MRS, which has a substantially different lineshape model, baseline model, and no regularisation, shows a similar, though lower magnitude bias for the default analysis case. As with the other algorithms, the preprocessing linewidth matching substantially reduces the bias. However, there is nearly no effect of the basis linewidth matching in FSL‐MRS.

A more detailed comparison between ABfit‐reg and LCModel is shown in Figure S9 for glutamate, with both approaches showing strong agreement. Comparing different dynamic block sizes (2, 5, 10) shows similar median levels of BOLD lineshape bias between 3 and 7 T; however, a clear increase in variance is apparent for the 3 T results—particularly for the results derived from 10 blocks. This increased variance at 3 T indicates that the bias may be less reliably detected for smaller sized studies (*N* = 20) due to metabolite estimation errors being dominated by low SNR rather than linewidth bias.

Table 2 lists the percentage bias for glutamate across the different field strengths and analysis methods. Default analyses gave a bias of around 1% for both LCModel and ABfit‐reg independent of field strength. Both preprocessing and basis‐based linewidth matching approaches reduced the bias to less than 0.09%, which is small enough to be insignificant for typical fMRS designs where glutamate is typically expected to change by at least 4% [1].

**TABLE 2:** A summary of glutamate bias for the conventional fitting methods, linewidth matching approaches and field strengths investigated. Data were preprocessed into five dynamic blocks before fitting.

Fit method	BOLD linewidth matching approach	Field strength	Mean Glu % change (|Cohen's *d*|)
LCModel	None	3 T	1.00 (0.85)
LCModel	None	7 T	1.03 (1.83)
LCModel	Preprocessing	3 T	0.06 (0.05)
LCModel	Preprocessing	7 T	0.00 (0.00)
LCModel	Basis	3 T	−0.03 (0.02)
LCModel	Basis	7 T	0.05 (0.09)
ABfit‐reg	None	3 T	0.87 (0.73)
ABfit‐reg	None	7 T	0.89 (1.64)
ABfit‐reg	Preprocessing	3 T	0.05 (0.04)
ABfit‐reg	Preprocessing	7 T	−0.02 (0.03)
ABfit‐reg	Basis	3 T	−0.08 (0.07)
ABfit‐reg	Basis	7 T	−0.09 (0.18)
FSL	None	3 T	0.30 (0.35)
FSL	None	7 T	0.31 (1.14)
FSL	Preprocessing	3 T	0.06 (0.07)
FSL	Preprocessing	7 T	−0.02 (0.07)
FSL	Basis	3 T	0.30 (0.35)
FSL	Basis	7 T	0.32 (1.16)

### 3.2 Dynamic Fitting

**FIGURE 3:**
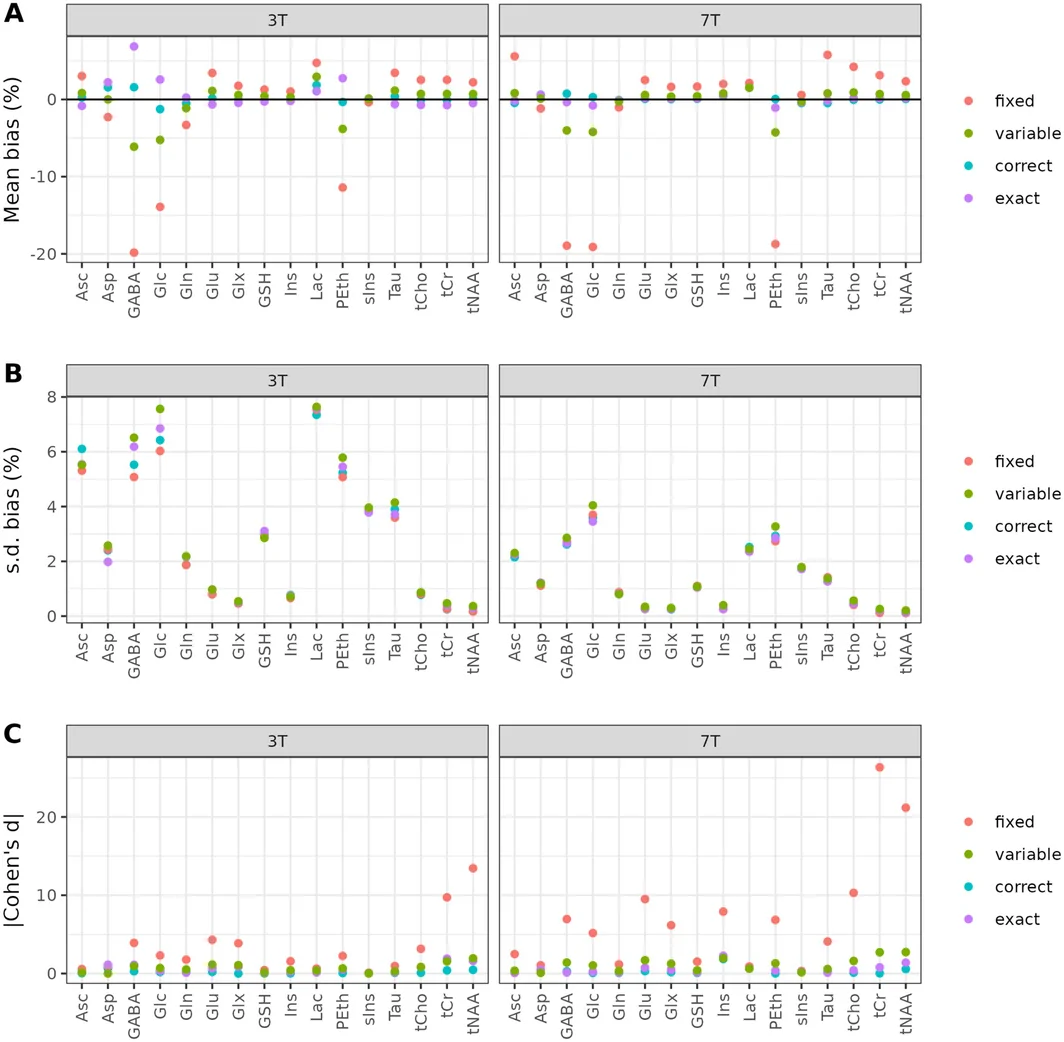
Strip plot of dynamic fitting‐measured metabolite concentration changes for each linewidth fitting model during the period of line narrowing. (A) Effect size as a percentage of the estimated constant concentration of the metabolite. The horizontal line is the ground truth zero change. (B) Standard deviation of the bias across all subjects (expressed as a percentage of the constant concentration). (C) Expressed as the magnitude Cohen's *d*.

The results of the dynamic fitting are presented as effect sizes (percentage relative to estimated baseline), standard deviation and Cohen's *d* in Table 3 and Figure 3.

**TABLE 3:** A summary of glutamate bias for the dynamic fitting methods and field strengths investigated.

Fit method	Dynamic fit model	Field strength	Mean Glu % change (|Cohen's *d*|)
FSL‐MRS	Fixed	3 T	3.4 (4.3)
FSL‐MRS	Fixed	7 T	2.5 (9.5)
FSL‐MRS	Variable	3 T	1.1 (1.1)
FSL‐MRS	Variable	7 T	0.6 (1.7)
FSL‐MRS	Exact	3 T	−0.6 (0.6)
FSL‐MRS	Exact	7 T	0.2 (0.8)
FSL‐MRS	Correct	3 T	0.2 (0.2)
FSL‐MRS	Correct	7 T	0.1 (0.31)

The imposition of an (incorrect) constant linewidth value across all spectra in the *fixed* model results in the largest biases observed in all models (ranging from −20% to +6%), with glutamate and lactate in the 2%–5% range at both field strengths. At 3 T, 13 of 16 metabolites assessed had a Cohen's *d* larger than 1, compared with 14 of 16 at 7 T.

The *exact* model, which implemented the correct Lorentzian lineshape model but estimated the line narrowing magnitude from the data, showed substantially reduced bias. The overall range was −1% to 7% with Glu showing −0.6 and 0.2% bias and Lac 1.5 and 1.8% bias for 3 T and 7 T respectively. At 3 T, 12 of 16 metabolites assessed had a Cohen's *d* smaller than 1, compared with 14 of 16 at 7 T.

The *correct* model fixed the magnitude of the line narrowing to the ground truth value as well as implementing the correct lineshape model. This model provided the lowest bias with the maximum bias, 1.8% shown by Lac at 3 T. Glutamate bias was 0.2 (3 T) and 0.1% (7 T). At 3 T, 16 of 16 metabolites assessed had a Cohen's *d* smaller than 1, compared with 15 of 16 at 7 T, with the average being the lowest of all dynamic models.

### 3.3 Bias in Fitting Apodised Data

**FIGURE 4:**
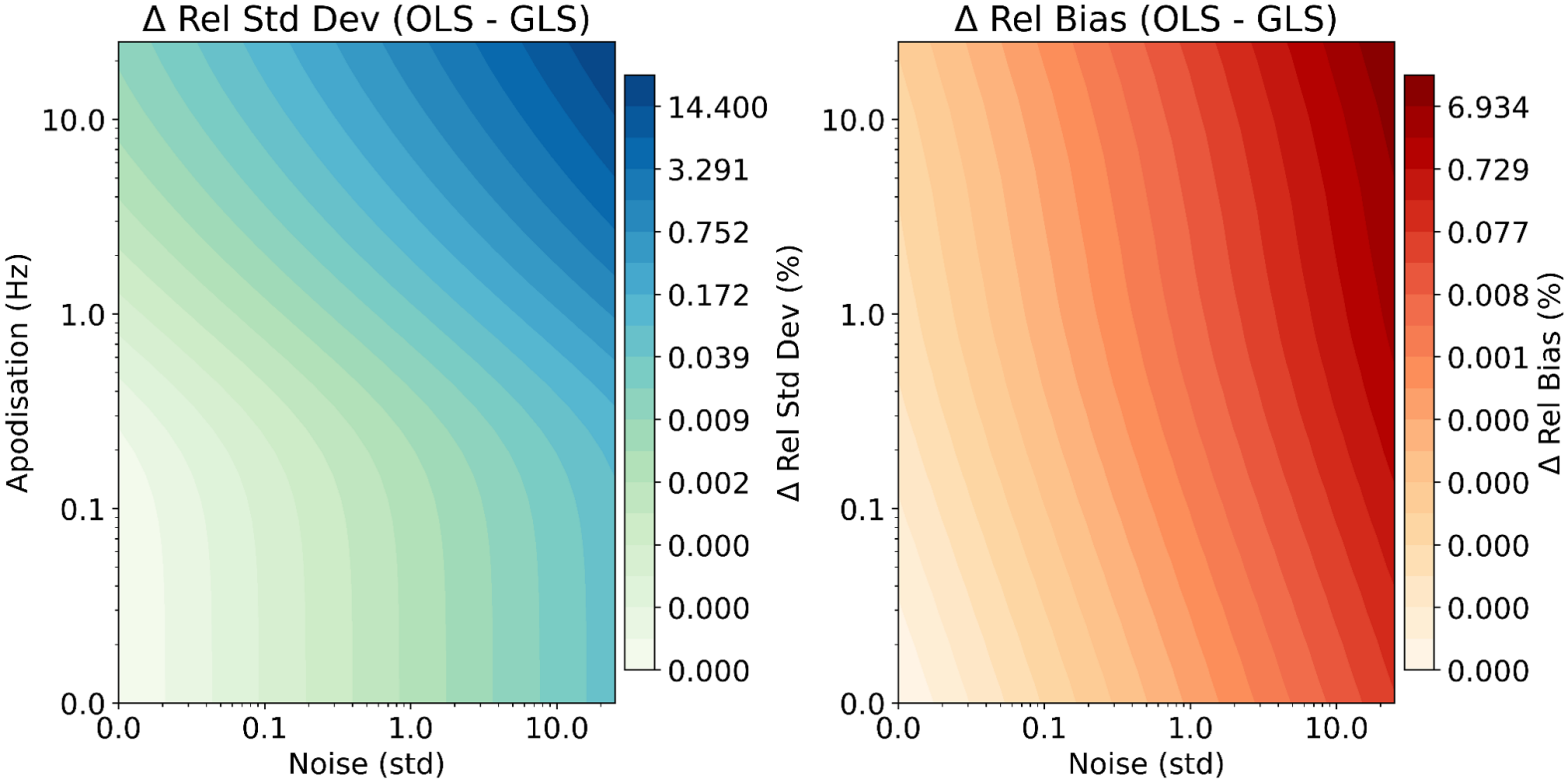
The percentage difference (relative to ground truth) between the fitted concentration standard deviation (left) and bias (right) predicted using the OLS and GLS asymptotic solutions. Results shown over a range of noise and apodisation magnitudes. Contours and axes both use a log scale for visualisation.

In Figure 4, we present percentage differences between the OLS and GLS predicted concentration bias and standard deviation over a wide range of noise and apodisation magnitudes. The absolute concentration bias and standard deviation for each individual method is presented in Figure S6. In the OLS case, both the biases, but predominantly the variance (and therefore standard deviation), increase with the apodisation. However, using GLS, the noise correlation is accounted for and the effect on the bias and variance remains consistent across apodisations; that is, there is no effect of linebroadening on the concentration. The difference between the OLS and GLS cases therefore informs us to what extent the apodised noise impacts the concentration estimate. We note that for both the OLS and GLS solutions, the primary impact of the noise is to increase the variance of the resulting distribution, because the impact on bias is asymptotically smaller. The concentration standard deviation is 0.01%, 0.03% and 0.55% larger than unbroadened data for 0.5, 1 Hz and 10 Hz broadening (OLS, noise = 1.0, equivalent to an unfiltered SNR of 40). Equivalently, the concentration bias is 0.003%, 0.005% and 0.017% larger than unbroadened data. The effect of apodisation on variance rises substantially with decreasing SNR; with a 10‐times lower SNR (of 4), the concentration standard deviation is equivalently 0.11%, 0.32% and 5.54% larger (than unbroadened data for 0.5, 1 and 10 Hz broadening).

## 4 Discussion

Overall, this study justifies the use of explicit BOLD lineshape modelling for conventional and dynamic fMRS analyses of brain activation at 3 T and above. Whilst the bias for conventional analyses is low (~1%) for commonly reported metabolites such as glutamate, a high proportion of studies (15 out of 35) summarised by Mullins et al. [1] reported a glutamate change of less than 5%, suggesting that BOLD bias is significant for certain fMRS studies. Explicit BOLD lineshape modelling is not commonly performed for fMRS at 3 T, yet our results show similar bias between 3 and 7 T. Whilst the BOLD lineshape change is approximately twice as large (in Hz) at 7 T compared with 3 T, the reduced spectral overlap and higher SNR at 7 T may counteract the increased bias, explaining our observations and strengthening the case for explicit BOLD lineshape modelling at 3 T.

Synthetic MRS is an important validation approach for MRS analysis research, because results may be compared directly to the ‘ground truth’; however, oversimplification of the simulation model risks producing results that do not generalise to experimental conditions. Limitations of this work include omitting the effects of subject movement, dynamic baseline instability and dynamic shimming instability. These factors were not considered to ensure any bias could be directly attributed to the BOLD lineshape modulation, and the good agreement between our estimates of glutamate bias and those reported from experimentally acquired fMRS at 7 T [4, 25] strongly support our simulation model assumptions.

A prolonged visual stimulation task was chosen for the simulated BOLD response due to this being the most replicated fMRS study; however, shorter ‘event‐related’ stimuli have also been used in fMRS studies [1]. The hemodynamic response function, and therefore BOLD response, is known to reach its maximum value for task durations of around 8 s and longer, with event‐related designs of 700 ms producing around 15% of this theoretical maximum. Despite this, a clear BOLD response has been observed for event‐related fMRS [18], suggesting it has the potential to introduce fitting bias. However, unlike prolonged tasks, the glutamate response to event‐related fMRS is thought to be much faster, peaking 500 ms after stimuli onset [1], whereas the BOLD response for a 700‐ms stimuli would take 5000 ms to reach a maximum. The reduced temporal similarity between the BOLD and glutamate responses for event‐related designs should significantly reduce the chances of confusion between the two processes; however, further work should involve improved characterisation of the glutamate response function combined with synthetic studies to confirm this hypothesis.

In this work, we assume the BOLD linewidth change is modelled with a purely Lorentzian character, arising from the assumption of a monoexponential signal decay associated with changes in tissue T2* relaxation [55]. This model is supported by several examples in the literature [4, 10, 33] where data from the active portion of the task are Lorentzian linebroadened to match the rest phase and subtracted. These subtraction spectra show very low levels of signal from the primary singlet metabolites (tNAA, tCr and tCho) whilst maintaining signal from task‐related metabolites such as lactate and glutamate, supporting the assumption of a Lorentzian lineshape change. A similar procedure with a purely Gaussian character would produce much larger residuals; however, we cannot rule out a small non‐Lorentzian contribution to the lineshape change and this area warrants further study.

Lorentzian lineshape matching is the most commonly applied approach to mitigating bias introduced by BOLD. It is achieved by applying a linebroadening apodisation filter to the time‐domain signal of the parts of the dataset thought to be affected by BOLD. This work has demonstrated, through an asymptotic framework, that fitting after data apodisation increases both fitting bias and standard deviation. At the level of linebroadening needed to mitigate BOLD at 3 or 7 T, that is, less than 1 Hz, the additional bias and standard deviation introduced are negligible (both < 0.1%). With greater BOLD effect at higher proposed field strengths, which at the time of writing include 14 T, requiring greater apodisation, or with lower SNR data, arising from the smaller voxels of MRSI, the effect of might not be negligible. The same asymptotic framework was used to validate that the use of GLS fitting removes the effect of apodisation on the fitting. GLS may potentially also remove the need to add additional noise as proposed in reference [4].

In this work, we compared the conventional Lorentzian lineshape matching step with a similar approach where the dynamic BOLD lineshape adjustment was applied to the basis‐set—rather than the synthesised spectral data. Figure 2 and Table 2 show a similar level of improvement for both approaches, for two of the fitting methods, suggesting either would be suitable for use for ABfit or LCModel. However, we note that additional investigation with experimentally acquired data would be required before assuming this is true in the general case. Whilst it may be initially surprising that these steps offer such a reduction in bias, we note that spectral fitting is a complex process, dependent on initialisation choices and with many non‐linear parameters. When coupled with the small changes (< 5%) associated with some fMRS paradigms, these biases have the potential to have a significant effect on any estimated changes in metabolite levels. Incorporation of prior knowledge of the BOLD time‐course acts to mitigate this bias in a way that is more reliable than conventional fitting, where the lineshape of each time point is estimated independently.

As a result of this work, we propose the following recommendations for mitigating BOLD‐derived biases in fMRS data:

1. That modelling or mitigating BOLD‐related linewidth change should be done at both 3 and 7 T.
2. That either preprocessing apodisation or basis linewidth matching is an appropriate way of mitigating bias when using LCModel and ABFit.
3. For FSL‐MRS, dynamic fitting should be used with an explicit BOLD model for linewidth. The magnitude of the BOLD‐related linewidth change can be directly estimated from the data in fitting (as in the exact case) or potentially pre‐calculated from fits to block‐averaged data (mimicking the correct approach).
4. For other fitting algorithms (including conventional use of FSL‐MRS), the preprocessing apodisation, with SNR matching, is recommended. This approach is immediately available to all users of fMRS.

We would emphasise that these recommendations are based on a synthetic dataset and that they should be implemented with care on experimental fMRS datasets.

This work is not constructed to compare fitting methods directly. However, it is clear that the different implementation and model choices across the methods (between software packages, or conventional versus dynamic fitting) do have an effect on bias observed. Firstly, FSL‐MRS, when used conventionally on these data, has lower bias than the other algorithms. This bias further drops if a different baseline model (no baseline) is used with FSL's conventional modelling. Rather than indicating any robustness on the part of FSL‐MRS, we propose that this is a result of the different lineshape and baseline model. Specifically, in this work, FSL‐MRS assumes all metabolite signals share a common Lorentzian lineshape broadening parameter, whereas LCModel and ABfit‐reg allow each metabolite to have an independent Lorentzian lineshape broadening. Greater lineshape freedom will reduce bias from metabolite T2 variability, expected in vivo, but this likely comes at the expense of increased bias from BOLD lineshape changes. This difference in model freedom is further exemplified by FSL‐MRS's bias not being corrected by the basis linewidth matching approach, which can likely be ascribed to one of FSL's specific initialisation approach, the absence of regularisation, the simpler lineshape model or a combination of these.

We also note that the dynamic fitting approach, as currently implemented, resulted in generally larger biases than the conventional methods. After investigation, we propose that this results from the model initialising from individually lower SNR per‐transient fits. Specifically, when the dynamic model is initialised with the true (ground‐truth) simulation parameters, the bias drops to levels similar to conventional methods. This suggests that there is no fundamental sensitivity penalty to dynamic fitting, but that careful initialisation is needed.

In conclusion, our results support the use of explicit BOLD lineshape correction or modelling for task‐based fMRS analysis to reduce fitting bias. Crucially, we show for the first time these steps are equally necessary for 3 T fMRS as 7 T, despite their infrequent use at 3 T. We have therefore made recommendations on how current practice could be updated to incorporate their use in standard fMRS analysis pipelines.

## Author Contributions

**Martin Wilson:** writing – original draft, writing – review and editing, conceptualisation, methodology, software, formal analysis, visualisation. **Simon M. Finney:** writing – review and editing, conceptualisation, methodology, software, formal analysis, visualisation. **William T. Clarke:** writing – review and editing, conceptualisation, methodology, software, formal analysis, visualisation.

## Funding

WTC and SF are funded by Wellcome (225924/Z/22/Z). The Centre for Integrative Neuroimaging was supported by core funding from the Wellcome Trust (203139/Z/16/Z and 203139/A/16/Z). For the purpose of open access, the author has applied a CC BY public copyright licence to any Author Accepted Manuscript version arising from this submission.

## Conflicts of Interest

The authors declare no conflicts of interest.

## Supporting information


**TABLE S1:** Molecular signals and associated concentrations used to generate synthetic MRS data.
**FIGURE S1:** Representative synthetic spectra for a single subject (A) single shot 3 T; (B) dynamic average (N = 640) 3 T; (C) single shot 7 T; (D) dynamic average (N = 320) 7 T.
**FIGURE S2:** Spectrograms illustrating the Lorentzian lineshape and noise matching preprocessing step with dashed horizontal lines representing the boundaries between the REST–TASK–REST–TASK–REST states. (A) Unprocessed spectra. (B) Unprocessed spectra with the dynamic mean spectrum subtracted. (C) Lineshape matched spectra with the dynamic mean spectrum subtracted. (D) Lineshape and noise matched spectra with the dynamic mean spectrum subtracted. Data were synthesised with the 7 T parameters listed in the main manuscript.
**FIGURE S3:** Example single peak spectra from each condition examined for spectral fitting bias after apodisation. The matched filter SNR for each case is printed in the top left. The single peak is Lorentzian (FWHM = 5 Hz) and on‐resonance (centred at 0 Hz).
**FIGURE S4:** Comparison of concentration, shift and line‐width distributions between the Monte Carlo (MC) and analytical predictions for both OLS and GLS optimisations with a noise standard deviation of one and an apodisation of 20 Hz.
**FIGURE S5:** Comparison of the NRMSE (%) between the Monte Carlo and analytical predictions for both OLS and GLS optimisations over a range of noise standard deviations and apodisation magnitudes.
**FIGURE S6:** The fitted concentration standard deviation (top) and bias (bottom) predicted using the OLS (left) and GLS (right) asymptotic solutions as a percentage of the ground truth value. Results shown over a range of noise and apodisation magnitudes. Contours and axes both use a log scale for visualisation.
**FIGURE S7:** BOLD lineshape–induced bias for commonly measured metabolites at 3 and 7 T. The LCModel analysis method was used to analyse five dynamic blocks (REST–TASK–REST–TASK–REST), and three analysis approaches were compared. Bias was calculated by evaluating the mean of the TASK blocks as a percentage change from the mean of the REST blocks on a participant by participant basis.
**FIGURE S8:** BOLD lineshape–induced bias for commonly measured metabolites at 3 and 7 T using the FSL‐MRS package. The FSL‐MRS analysis method was used to analyse five dynamic blocks (REST–TASK–REST–TASK–REST), and three analysis approaches were compared. Bias was calculated by evaluating the mean of the TASK blocks as a percentage change from the mean of the REST blocks on a participant by participant basis.
**FIGURE S9:** Box and whisker plots of the percentage change in glutamate level between the REST and TASK conditions. Field strength, number of dynamic blocks, fitting method and BOLD linewidth correction approaches are shown for comparison.

## Data Availability

The data that support the findings of this study are openly available in Zenodo at https://doi.org/10.5281/zenodo.18873756. The code required to run all simulations is available online in two repositories. For synthetic data generation and conventional fitting: https://github.com/martin3141/fmrs_bold_bias_paper and for the dynamic fitting approaches: https://github.com/wtclarke/fmrs_bold_dynamic_fitting (commit e289dbab9c9bc87f53b4f9029714fd88cfcbcba6).

## Appendix A Asymptotic Derivation of the Effect of Lorentzian Broadening on Spectral Fitting

Problem Summary

Here, we asymptotically derive the impact of Lorentzian broadening (apodisation) on the bias and variance of model parameters through both ordinary least squares (OLS) and generalised least squares (GLS) spectral fitting. Below, we define the cost function for both least squares methods, the asymptotic expansion around the true parameter values and finally the leading‐order bias and variance under this asymptotic expansion. We then present a simple forward model for spectral fitting (single Lorentzian peak) as well as analytical expressions for the Jacobian and Hessian. Finally, we analytically derive the noise covariance matrix in both the time and spectral domains when apodised by a spectral linebroadening window. Validation of the asymptotic solutions via comparison with Monte Carlo simulations is present in Figures S3–S5.

The Model and Cost Function

Let the observed complex data be Z, generated by the true parameters $\theta_{0}$,

$$Z=M\left(\theta_{0}\right)+\epsilon ,$$

M is a general forward model, and the noise is complex circular Gaussian, $\epsilon ∼N_{C}\left(0,\Sigma \right)$.

We seek the estimate $\hat{\theta }=\theta_{0}+\Delta \theta$ that minimises the generalised cost function with a weighting matrix Ω:

(1)
$$C\left(\theta \right)={\left(Z-M\left(\theta \right)\right)}^{H}\Omega \left(Z-M\left(\theta \right)\right).$$

For now, we will keep the weighting matrix general; however, we note that for OLS, Ω=I and for GLS $\Omega =\Sigma^{-1}$, where Σ is the known noise covariance matrix (derived later). Setting the gradient of Equation (1) with respect to θ to zero at $\hat{\theta }$ gives

(2)
∇Cθ^=−2ReJθ^HΩZ−Mθ^=0,

where $J\left(\theta \right)=\nabla_{\theta }M\left(\theta \right)$ is the Jacobian.

Asymptotic Expansion

We proceed by defining the asymptotic parameter as the typical magnitude of the noise ϵ~∣ϵ∣. The asymptotic assumption holds when $\epsilon \ll M\left(\theta_{0}\right)$ such that the impact of the apodised noise may be considered a perturbation on the true parameter values, $\theta_{0}$. It will be convenient to expand $\hat{\theta }$ to second order,

(3)
$$\begin{array}{c} \hat{\theta }=\theta_{0}+\epsilon \theta_{1}+\epsilon^{2}\theta_{2}+O\left(\epsilon^{3}\right).\; \end{array}$$

Here, $\theta_{i}$ is the ith order contribution to the asymptotic approximation of $\hat{\theta }$. Because ϵ is small, each consecutive term in this expansion is less impactful on the resulting solution. The total error in the estimate may be recovered via $\Delta \theta =\epsilon \theta_{1}+\epsilon^{2}\theta_{2}+O\left(\epsilon^{3}\right)$.

Using the expansion of $\hat{\theta }$ in Equation (3), we Taylor expand $M\left(\hat{\theta }\right)$ and $J\left(\hat{\theta }\right)$ around the true parameters $\theta_{0}$. Let $J=J\left(\theta_{0}\right)$ and let $H=\nabla_{\theta }^{2}M\left(\theta_{0}\right)$ be the Hessian tensor.

The expansions up to second order in ϵ are

$$\begin{array}{c} M\left(\hat{\theta }\right)\approx M\left(\theta_{0}\right)+\mathrm{\epsilon J}\theta_{1}+\epsilon^{2}J\theta_{2}+\frac{1}{2}\epsilon^{2}\theta_{1}^{T}H\theta_{1},\\ Z-M\left(\hat{\theta }\right)\approx \epsilon -\mathrm{\epsilon J}\theta_{1}-\epsilon^{2}J\theta_{2}-\frac{1}{2}\epsilon^{2}\theta_{1}^{T}H\theta_{1},\\ J\left(\hat{\theta }\right)\approx J+\epsilon \theta_{1}\cdot H, \end{array}$$

where · denotes tensor contraction over the parameter dimension. Substituting these into Equation (2) yields

(4)
0=ReJ+ϵθ1·HHΩϵ−ϵJθ1ϵ2Jθ2−12ϵ2θ1THθ1.

First‐Order Error and Variance

We aim to asymptotically predict the bias $E\left[\Delta \theta \right]$ and variance $E\left[\Delta \theta \Delta \theta^{T}\right]$ of predicted offset due to the apodised noise. To do this, we first consider the first‐order terms (linear in ϵ). Extracting the $O\left(\epsilon \right)$ terms from Equation (4) gives

0=ReJHΩϵ−JHΩJθ1.

Solving for the first‐order error:

(5)
θ1=ReJHΩJ−1ReJHΩϵ=MΩReJHΩϵ,

where MΩ=ReJHΩJ−1 is the unweighted projection matrix. Because $E\left[\epsilon \right]=0$, the predicted first‐order bias is zero. The parameter variance is given by

(6)
$$\begin{array}{c} V_{\Omega }=E\left[\theta_{1}\theta_{1}^{T}\right]. \end{array}$$

Substituting Equation (5) into Equation (6), using the identity

ERexRexT=12ReExxH+ExxT,

and assuming ReEϵϵT=0 we obtain

(7)
VΩ=MΩReJHΩΣΩJMΩ,

where $\Sigma =E\left[\epsilon \epsilon^{H}\right]$ is the noise covariance matrix.

Because the first‐order bias is zero (and the first‐order variance non‐zero), this suggests that the primary impact of the apodised noise on the resulting distribution is to increase in variance. This is confirmed in the later comparison with MC simulations.

Second‐Order Error and Bias

In order to calculate the leading‐order impact of the apodised noise on the parameter estimation bias, we must consider the second‐order asymptotic problem (terms quadratic in ϵ). Extracting the $O\left(\epsilon^{2}\right)$ terms from Equation (4) gives

0=Re−JHΩJθ2−12JHΩθ1THθ1+θ1·HHΩϵ−θ1·HHΩJθ1.

Solving for $\theta_{2}$ and taking the expectation $b=E\left[\theta_{2}\right]$ yields the leading‐order asymptotic bias,

bΩ=−12MΩReJHΩEθ1THθ1+MΩReEθ1·HHΩϵ−Eθ1·HHΩJθ1.

It may be shown that this reduces to

(8)
bΩ=−12MΩReJHΩH:VΩ+MΩRe12ΩΣΩJMΩ−ΩJVΩ,

where: is a contraction between tensors.

OLS Versus GLS

For OLS (not whitened), we substitute Ω=I into the general equations for the variance and bias (Equations 7 and 8). Simplifying, we obtain the leading order equations

(9)
VU=12MUReJHΣJMU,whereMU=ReJHJ−1,bU=−12MUReJHH:VU+MURe12ΣJMU−JVU.

Conversely, for GLS (whitened residual), we substitute $\Omega =\Sigma^{-1}$ into Equations (7) and (8) to obtain

(10)
VW=12MW=12ReJHΣ−1J−1,bW=−12MWReJHΣ−1H:VW.

We note that the last term in Equation (8) cancels for GLS.

Lorentzian Model

We now present a simple forward model to test the impacts of apodisation, a single Lorentzian peak defined by a concentration, shift and line‐width. The Jacobian and Hessian may be substituted into the above expressions for the bias and variance (Equations 9 and 10).

Definitions and Base Terms

We define the parameter vector as $x={\left[c,s,\gamma \right]}^{⊤}$, where c is the concentration, s is the frequency shift and γ is the linebroadening.

Let $b\left(t\right)$ represent the original basis function in the time domain. The parametrised time‐domain basis signal $m\left(t\right)$ is given by:

$$m\left(t\right)=\exp\left(-\left(\text{is}+\gamma \right)t\right)b\left(t\right)$$

To simplify the notation for the frequency‐domain signal and its derivatives, let the Fourier transform (F) of the time‐weighted signals be defined as $F_{k}$:

$$\begin{array}{c} F_{0}=F\left\{m\left(t\right)\right\}\\ F_{1}=F\left\{t\cdot m\left(t\right)\right\}\\ F_{2}=F\left\{t^{2}\cdot m\left(t\right)\right\} \end{array}$$

1 Forward Model (S)

The frequency‐domain forward model S is the concentration scaled by the zeroth‐order Fourier transform of the basis:

$$S\left(c,s,\gamma \right)=c\cdot F_{0}$$

2 Jacobian (J)

The Jacobian is the matrix of first‐order partial derivatives of the forward model with respect to each parameter. For a single spectral point, it is a 1×3 row vector:

$$J=\left[\begin{array}{ccc} \frac{\partial S}{\partial c} & \frac{\partial S}{\partial s} & \frac{\partial S}{\partial \gamma } \end{array}\right]$$

Evaluating the partial derivatives of S yields the final Jacobian vector:

$$J=\left[\begin{array}{ccc} F_{0} & -\mathrm{ic}F_{1} & -cF_{1} \end{array}\right]$$

3 Hessian (H)

The Hessian is the 3×3 symmetric matrix of second‐order partial derivatives:

$$H=\left[\begin{array}{ccc} \frac{\partial^{2}S}{\partial c^{2}} & \frac{\partial^{2}S}{\partial c\partial s} & \frac{\partial^{2}S}{\partial c\partial \gamma }\\ \frac{\partial^{2}S}{\partial s\partial c} & \frac{\partial^{2}S}{\partial s^{2}} & \frac{\partial^{2}S}{\partial s\partial \gamma }\\ \frac{\partial^{2}S}{\partial \gamma \partial c} & \frac{\partial^{2}S}{\partial \gamma \partial s} & \frac{\partial^{2}S}{\partial \gamma^{2}} \end{array}\right]$$

Calculating the individual second‐order derivatives and populating the symmetric matrix gives

$$H=\left[\begin{array}{ccc} 0 & -iF_{1} & -F_{1}\\ -iF_{1} & -cF_{2} & \mathrm{ic}F_{2}\\ -F_{1} & \mathrm{ic}F_{2} & cF_{2} \end{array}\right]$$

Spectral Noise Covariance in MRS After Lorentzian Broadening

Time Domain

We first define complex white Gaussian noise with N points in the time domain:

(11)
$$\begin{array}{c} E\left[\nu_{n}\right]=0,\;\;\text{such that}\;\;E\left[\nu_{n}\nu_{m}^{*}\right]=\sigma_{t}^{2}\;\delta_{\mathrm{nm}}, \end{array}$$

$\sigma_{t}^{2}$ is the time‐domain variance. We define the apodised (filtered) time‐domain noise as

$$\nu_{n}^{\prime }=\nu_{n}W_{n},$$

where $W_{n}$ is the purely real, time‐domain filter (window).

Frequency Domain

In the frequency (or spectral) domain, the apodised (filtered) noise is the discrete Fourier transform (DFT) of $\nu_{n}^{\prime }$:

$${\tilde{\nu }}_{k}=\sum_{n=0}^{N-1}W_{n}\nu_{n}\;e^{-i2\mathrm{\pi kn}/N}.$$

Definition of Covariance.

The covariance of the apodised noise in the spectral domain is then

$$\mathrm{Cov}\left({\tilde{\nu }}_{k},{\tilde{\nu }}_{\ell }\right)=E\;\left[\left(\sum_{n=0}^{N-1}W_{n}\nu_{n}e^{-i2\mathrm{\pi kn}/N}\right)\left(\sum_{m=0}^{N-1}W_{m}\nu_{m}e^{i2\pi \ell m/N}\right)\right].$$

Exploiting the linearity of the summation and expectation operators, and using Equation (11), we find

$$\mathrm{Cov}\left({\tilde{\nu }}_{k},{\tilde{\nu }}_{\ell }\right)=\sum_{n=0}^{N-1}\sigma_{t}^{2}\;W_{n}^{2}\;e^{-i2\mathrm{\pi n}\left(k-\ell \right)/N}$$

such that the covariance is the scaled Fourier transform of the square of the time‐domain window:

$$\mathrm{Cov}\left({\tilde{\nu }}_{k},{\tilde{\nu }}_{\ell }\right)=\sigma_{t}^{2}\times \mathrm{DFT}\left\{W_{n}^{2}\right\}\;\mathrm{at}\;\text{frequency index}\;\left(k-\ell \right),$$

Derivation of the Spectral Noise Covariance for a Linebroadening Window

Consider the discrete‐time window:

$$W_{n}=e^{-\mathrm{\gamma n}},\;\;n=0,1,\ldots ,N-1,$$

where γ>0, is the Lorentzian linebroadening to be applied.

DFT Definition:

Applying an N‐point DFT to $W_{n}^{2},$ we have

$$X_{k}=\sum_{n=0}^{N-1}e^{-2\mathrm{\gamma n}}e^{-i\frac{2\pi }{N}\mathrm{kn}}$$

which may be expressed in closed form as the finite geometric series,

$$X_{k}=\frac{1-e^{-2\mathrm{\gamma N}}}{1-e^{-2\gamma }e^{-i\frac{2\pi }{N}k}}.$$

The final covariance matrix can be written as a circulant matrix substituting in the expression $X_{k}$

$$\left(\begin{array}{ccccc} X_{N/2} & X_{N/2+1} & X_{N/2+2} & \cdots & X_{N/2-1}\\ X_{N/2-1} & X_{N/2} & X_{N/2+1} & \cdots & X_{N/2-2}\\ X_{N/2-2} & X_{N/2-1} & X_{N/2} & \cdots & X_{N/2-3}\\ \vdots & \vdots & \vdots & \ddots & \vdots \\ X_{N/2+1} & X_{N/2+2} & X_{N/2+3} & \cdots & X_{N/2} \end{array}\right).$$
